# Current and Future Trends in Molecular Biomarkers for Diagnostic, Prognostic, and Predictive Purposes in Non-Melanoma Skin Cancer

**DOI:** 10.3390/jcm9092868

**Published:** 2020-09-04

**Authors:** Taxiarchis Konstantinos Nikolouzakis, Luca Falzone, Konstantinos Lasithiotakis, Sabine Krüger-Krasagakis, Alexandra Kalogeraki, Maria Sifaki, Demetrios A. Spandidos, Emmanuel Chrysos, Aristidis Tsatsakis, John Tsiaoussis

**Affiliations:** 1 Laboratory of Anatomy-Histology-Embryology, Medical School, University of Crete, 71110 Heraklion, Crete, Greece; medp2011826@med.uoc.gr; 2 Department of General Surgery, University General Hospital of Heraklion, 71110 Heraklion, Crete, Greece; k.lasithiotakis@uoc.gr (K.L.); manolischrysos@gmail.com (E.C.); 3 Epidemiology Unit, IRCCS Istituto Nazionale Tumori ‘Fondazione G. Pascale’, I-80131 Naples, Italy; l.falzone@istitutotumori.na.it; 4 Dermatology Department, University Hospital of Heraklion, 71110 Heraklion, Crete, Greece; krkras@med.uoc.gr; 5 Department of Pathology-Cytopathology, Medical School, University of Crete, 70013 Heraklion, Crete, Greece; a.kalogeraki@med.uoc.gr; 6 Centre of Toxicology Science and Research, Faculty of Medicine, University of Crete, 71003 Heraklion, Crete, Greece; skinclinicsifaki@gmail.com; 7 Laboratory of Clinical Virology, Medical School, University of Crete, 71003 Heraklion, Crete, Greece; spandidos@spandidos.gr

**Keywords:** non-melanoma skin cancer, basal cell carcinoma, squamous cell carcinoma, Merkel cell carcinoma, telomeres, telomerase, epigenetics, miRNA

## Abstract

Skin cancer represents the most common type of cancer among Caucasians and presents in two main forms: melanoma and non-melanoma skin cancer (NMSC). NMSC is an umbrella term, under which basal cell carcinoma (BCC), squamous cell carcinoma (SCC), and Merkel cell carcinoma (MCC) are found along with the pre-neoplastic lesions, Bowen disease (BD) and actinic keratosis (AK). Due to the mild nature of the majority of NMSC cases, research regarding their biology has attracted much less attention. Nonetheless, NMSC can bear unfavorable characteristics for the patient, such as invasiveness, local recurrence and distant metastases. In addition, late diagnosis is relatively common for a number of cases of NMSC due to the inability to recognize such cases. Recognizing the need for clinically and economically efficient modes of diagnosis, staging, and prognosis, the present review discusses the main etiological and pathological features of NMSC as well as the new and promising molecular biomarkers available including telomere length (TL), telomerase activity (TA), CpG island methylation (CIM), histone methylation and acetylation, microRNAs (miRNAs), and micronuclei frequency (MNf). The evaluation of all these aspects is important for the correct management of NMSC; therefore, the current review aims to assist future studies interested in exploring the diagnostic and prognostic potential of molecular biomarkers for these entities.

## 1. Introduction

Skin cancer is currently the most common type of cancer among Caucasians [1]. It is estimated that approximately 1 in 5 Americans will develop skin cancer at some point in their lives by the age of 70 [2]. Unfortunately, in spite of immense efforts being made in public health awareness and primary prevention campaigns, a steady increase in skin cancer rates is observed [3,4,5]. In fact, non-melanoma skin cancer (NMSC) is the most common type with a relative incidence increase of up to 10% per annum, with 2–3 million new cases each year globally [6]. Skin cancer includes several distinct subtypes which can be divided in two main categories, malignant melanoma, and NMSC, with the latter being further divided into basal cell carcinoma (BCC), cutaneous squamous cell carcinoma (cSCC), Bowen disease (BD), actinic keratosis (AK), and Merkel cell carcinoma (MCC) each of which has a different biological behavior, etiology, and prognosis [6]. From these five distinct entities, BCC, SCC, and MCC stand out, given their potential to invade into deeper layers and metastasize [7,8].

BD is in nature an in situ SCC, while AK is a precancerous lesion acting as precursor to SCC. Even though they both exhibit a close association with SCC, they present different histopathological findings [8]. BCCs are more benign lesions having an almost absent metastatic potential, whereas SCCs exhibit a metastatic risk between 0.1–13.7% [9]. Given the fact that the global population is aging, an increase in the associated morbidity and local recurrence rates is to be expected. This in hand creates a great burden on national healthcare systems and economies. Accounting for 70–80% of all skin cancer cases, BCC is ranked among the most common types of cancer [10]. However, given the benign nature of BCCs and the ease of treatment in a doctors’ office, the majority of cases are not recorded in most national cancer registries [11]. BCC preferentially arises from stem cells within hair follicles and mechanosensory niches [12]. Generally, BCC is a slow-growing tumor which rarely gives rise to distant metastases. However, if left untreated, it can grow invasively, destroying underlying tissues. It has been shown that patients with BCC face a 10-fold risk of developing another BCC compared to the general population [13]. Nonetheless, given its benign character, no long-term follow-up is required following a complete resection of the primary tumor [14]. SCC is the second most frequent type of skin cancer [3]. As already mentioned, SCC usually occurs on sun-exposed areas of the skin, such as the head, face, earlobe, lips, or torso. Nonetheless, it can also arise from the surrounding skin of the anus and genitalia, or even from skin with chronic inflammation, such as a scar or chronic wound [15]. If left untreated, an in situ SCC (AK or BD) may evolve into an invasive SCC with a great risk of metastasizing or relapsing [16].

MCC is a rare type of NMSC arising from Merkel cells. Epidemiological findings have identified UV radiation, old age, male sex, and Caucasian descent as strong risk factors contributing to the surprising increase in incidence rates by 95% between 2000 and 2013 [17]. In cases with immunosuppression in particular, an aggressive form is exhibited with mortality rates exceeding 30% [18,19,20,21]. However, the pathophysiology of MCC development is not yet fully understood. Under poorly understood circumstances, Merkel cells produce the neuroendocrine lesion termed MCC. From its early discovery in 1972 by Toker [22], MCC has changed several names some of which are “cutaneous neuroendocrine carcinoma”, “cutaneous trabecular carcinoma”, and “small cell primary cutaneous carcinoma” [23]. Various mechanisms have been suggested to induce Merkel cell carcinogenesis. such as cellular senescence, immunosuppression, and the potential oncogenic pathways induced by UV exposure (UV-specific mutations in the p53 gene) [24]. Recently, Feng et al. found that a novel type of polyoma virus may attribute to MCC formation [25], highlighting the increased complexity of this entity. Being a multifactorial disease, NMSC remains a challenge for clinicians and researchers, not only to understand its biological behavior, but also to develop better tailored and personalized treatment plans [26]. Fortunately, as proven from various national cancer registries, the majority of NMSC cases exhibit an excellent 5-year survival rate ranging from 100% for BCC to 95% for SCC [27,28] with local recurrence rates being < 5% [29,30]. It is clear that the early diagnosis of primary and relapsed tumors in addition to carefully tailored treatments will be greatly assisted from the introduction of appropriate biomarker panels into everyday clinical practice. Thus, the present brief review aims not only to introduce the clinical significance of using biomarkers for NMSC, but also to pinpoint novel biomarkers worthy of further research. From the great number of molecular biomarkers under research, we choose to present those which are most likely to be introduced into everyday clinical practice in the near future, such as the miRNAs, and those which according to the current literature are most promising candidates requiring further investigation.

## 2. Etiology

According to the current knowledge on NMSC development, a constellation of factors are found to be implicated such as environmental exposure to UV light (regions closer to the equator suffer from higher rates of NMSC) [5,31], radiotherapy [32], viral infections (mostly β-HPV) [3], immunosuppression (based primarily upon the increased incidence exhibited in organ transplant recipients and the twofold higher incidence rate among HIV+ patients where SCCs is positively correlated with immunosuppression) [33,34], and genetic predisposition [35].

### 2.1. Ultraviolet (UV) Light

UV light exposure has been found to result in DNA mutations by inducing covalent bonding between adjacent pyrimidines (from UVB) and the formation of reactive oxygen species (from UVA) [36]. In detail, NMSCs formation has been positively associated to recreational UV light exposure with 2.5- and 1.5-fold increase in the risk of developing SCC and BCC, respectively [37]. Moreover, prolonged sunlight exposure during childhood and adolescence has been found to be responsible for BCCs, while chronic UV exposure is SCC formation in more advanced ages [1]. Notably, UV light may have a carcinogenic effect via immunosuppression. In detail, it has been described that a cellular modulation of immune cells is evoked, as evidenced by the concomitant depletion of Langerhans cells from the epidermis, altered antigen presentation in the lymph nodes, a shift towards Th2 responses and the development of tumor antigen-specific T regulatory cells, resulting in blocked immune surveillance and tumor outgrowth [38,39,40].

### 2.2. Genetic Background

Genetic predisposition is neither present nor uniform across all NMSCs. Most BCCs lack any pre-existing genetic background while SCCs may arise from a genetically predisposed clonal cell growth. Genetic damage accumulates, leading first to precursor lesions of AK or BD and subsequently to SCC [6] allowing even for multifocal development of SCCs (field cancerization) [41,42]. Several tumor suppressor genes and proto-oncogenes have been found to be implicated in BCC pathogenesis, such as components of the Sonic Hedgehog pathway (PTCH1 and SMO), the TP53 tumor suppressor gene, and members of the RAS family. In fact, it seems that the improper activation of the Sonic Hedgehog pathway is the key component pathway in BCC carcinogenesis [43,44]. SCCs are also driven by several mutated genes [45]. In detail, several mutations of the tyrosine kinase receptors (epidermal growth factor receptor-EGFR and fibroblast growth factor receptors—FGFRs) [46], certain cell cycle regulatory genes (TP53-the most common somatic mutation, CDKN2A/RB1, CCDN1, and MYC) [47,48], the RAS/MAPK and PI3K signaling pathways [46], genomic loci implicated in squamous cell fate determination (TP63, SOX2, and NRF2) [49,50,51], and squamous differentiation network (Notch and Fat1) [52,53] have been found.

### 2.3. Infectious Agents

An increasing body of evidence highlights the oncogenic potential of certain viruses such as the HPV, EBV, and the recently discovered Merkel Cell Polyomavirus (MCPyV) for NMSCs. HPV produces the E6 and E7 oncoproteins which have the potential to integrate into the hosts’ keratinocytes genome [54,55]. It is worth noting that HPV-positive NMSC presents a more benign clinical behavior than HPV-negative NMSC. Even though the reason behind this remains undetermined, it may be due to the fact that the majority of the HPV-positive NMSCs tend to express wild-type TP53. On the counterpart, the majority of the HPV-negative cases exhibit mutated TP53 with or without accompanying mutations in other genomic loci [45]. On the contrary, EBV-induced carcinogenesis results from a multistep process, where the effect from a chronic EBV infection augments the results driven from genetic and epigenetic (methylation of several genomic sites and modulators) changes in the keratinocytes’ genome [56]. In 2008, Feng et al. identified the MCPyV [25]. Ever since, epidemiological studies using serological tests have estimated that 60% to 80% of the population is infected with MCPyV [57,58]. Interestingly, the majority of MCC cases (approximately 75%) are linked to MCPyV infection [59,60,61]. Even though p53 is considered to be a hallmark for NMSCs, Sihto et al. demonstrated that the upregulation of p53 is not a mandatory step for Merkel cell carcinogenesis. In fact, they found p53 to be overexpressed only in 7% of the MCPyV-positive MCC samples suggesting that MCPyV-associated carcinogenesis does not rely on the p53 pathway [62]. Based on the current literature, the proposed mode of MCPyV-induced carcinogenesis relies on at least two critical steps; integration of viral DNA into the cells’ genome and loss of its ability to replicate due to accumulated mutations. Following these two steps, the virus produces two main carcinogenic proteins; large T-antigen (LTAg) and small t-antigen (STAg) [62,63,64,65]. It has been shown that LTAg specifically binds to tumor suppressor proteins, including p53 (TP53) and members of the Rb family (RB1, RBL1, and RBL2) [66,67,68].

## 3. Current Molecular Biomarkers for NMSC

### 3.1. Telomere Length (TL)

**Figure 1:**
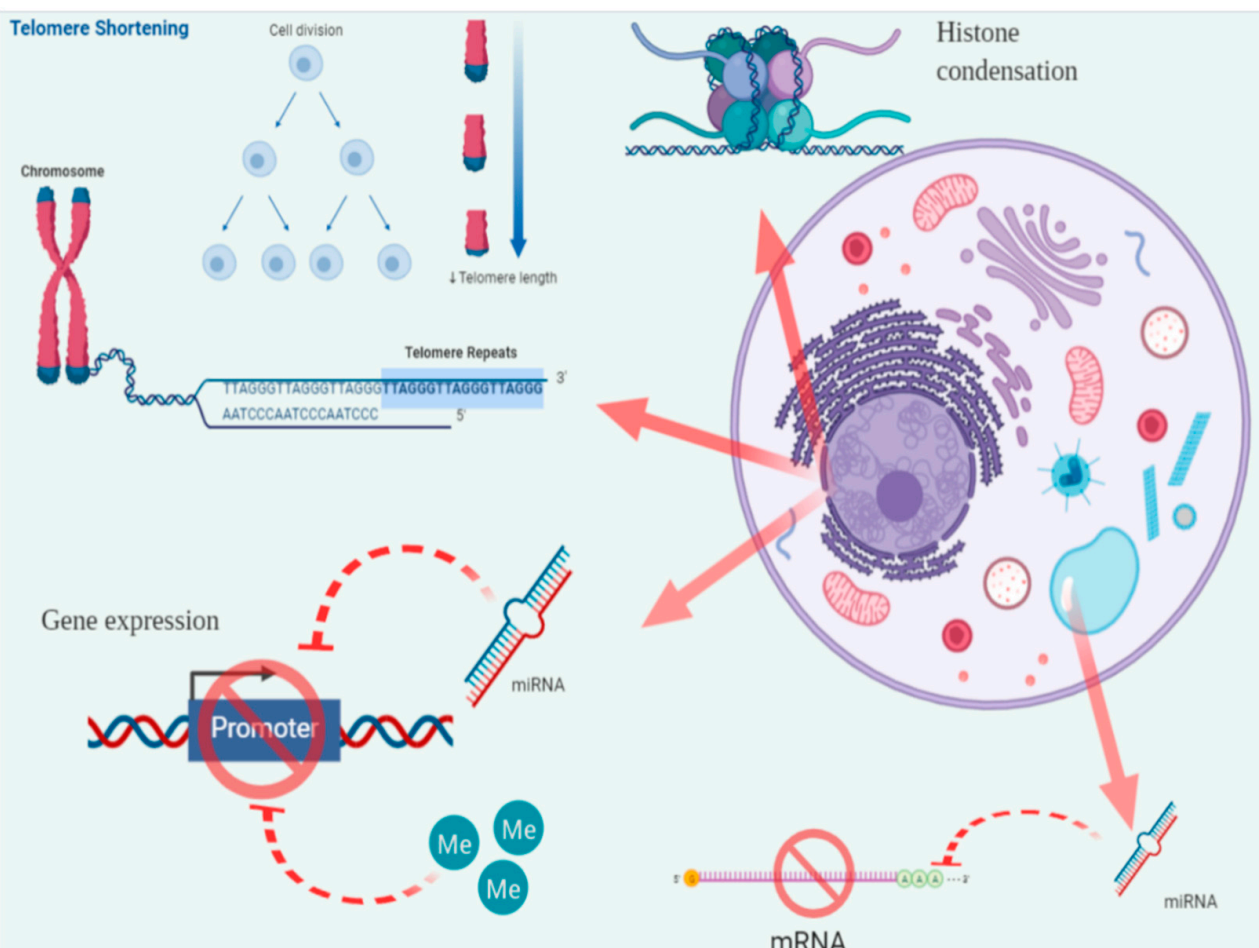
Graphic representation of the underlying pathophysiology of NMSC formation. Carcinogenic mechanisms located in the nuclear apartment involve telomere shortening, histone condensation, inactivation of tumor-suppressor promoters by miRNA and/or methylation. Carcinogenic mechanisms located in the cytosol involve inactivation of mRNAs by miRNAs. Me: methylation.

Telomeres are repetitive nucleotide sequences (5′-TTAGGG-3′) added on the ends of eukaryotic chromosomes by an enzyme, the telomerase. Combined with specific proteins, telomeres form complexes guarding chromosomic ends from degradation induced by repetitive cell divisions [69] and oxidative stress [70] (Figure 1). Telomerase is an enzyme complex consisting of the catalytic subunit, the human telomerase reverse transcriptase (hTERT) and an RNA template-hTR (human telomere RNA), the telomerase RNA component (TERC), which serves as a template for directing the appropriate telomeric sequences onto the 3′ end of a telomeric primer [71]. Given the well-established knowledge that shorter telomeres contribute to cellular senescence [72], both tTL and telomerase activity (TA) have been the subject of research on cancer-related biomarkers. In fact, an increasing body of evidence supports the potential of both serving as diagnostic and prognostic biomarkers for various cancers [73,74]. The underlying hypothesis is that when cellular senescence is combined with excessive environmental burden (for instance UV exposure), the cell may be led to apoptosis. Thus, in theory, it would be reasonable to expect neoplastic cells to possess longer telomeres. On the contrary though, shorter telomeres would render cellular DNA prone to mutations due to replication errors, leading to chromosomal instability and subsequent chromosomal aberrations and therefore, cancer [75]. Nonetheless, from what has been found, it seems that both scenarios may be true for the pathogenesis of NMSC [76], which could be the reason why such a great heterogeneity has been found in association studies [77].

Using Q-FISH for the determination of TL in neoplastic epidermal cells, Yamada-Hishida et al. found that TL was decreased in BD and AK (both had relatively close TL) in relation to BCC and SCC, suggesting that TL estimation in NMSC reflects its biological behavior, such as the metastatic and invasive potential. Moreover, the authors suggested that SCC precursor lesions exhibit a different TL from those of SCC [78]. On the contrary, Wainwright et al. examined BCC and TL in relation to normal skin and reported that telomeres from BCC samples had a variable range of TL (out of the 20 samples they examined 13, had an increased mean TL, while 7 had a shorter TL) [79]. A possible explanation for this variability may be the sampling variability. In other words, the fact that when testing TL from neoplastic cells, one has to bear in mind that cells at one point will differ from those at another despite their relative distance. A solution to this problem was indicated by Han et al., who presented that TL in peripheral blood lymphocytes (PBLs) can be indicative of the skin neoplastic burden and can thus be used as a biomarker. Of note, they found that there was no clear association between TL and the risk of SCC development. By contrast, a shorter telomere length was shown to be associated with an increased risk of BCC [80]. Another study supporting these results was published by Anic et al., who evaluated the relative risk of NMSC development in relation to TL in PBLs. They found that longer telomeres were negatively-associated with BCC and SCC formation (particularly in males), regardless of age [81]. In contrast to the above-mentioned studies, Liang et al. in an equally large series of NMSC cases, reported that there was no association between TL in PBLs and the risk of developing NMSC [82].

A rather interesting finding reporting the potential use of TL as a promising indicator of the underlying genetic background giving rise to SCC and the rest of the NMSC was published in the study by Leukfe et al. In detail, they presented that TL distribution is able to differentiate between two types of genetically distinct skin SCCs. The first type exhibits a short/homogeneous TL profile, while the other one a long/heterogeneous TL profile. According to the authors, these findings point out the possibility of two co-existing carcinogenic mechanisms. The first scenario suggests an epidermal stem cell that from some point exhibited accelerated telomere loss which was then passed to his daughter-cells. On the contrary, in the second scenario, which may be the case for the majority of skin SCC cases, a multifocal carcinogenic process occurs with variable proliferation rates at each site, which in hand give rise to variable TLs. In addition, this scenario may explain the profound genetic heterogeneity seen among cancer cells even from the same lesion [76]. This is also important for the determination of the high-risk precursor lesions whose TL resembles that of SCC. Recognizing such lesions would be important for the application of closer monitoring protocols, given that they are more likely to metastasize or recur.

### 3.2. Telomerase Activity (TA)

As mentioned above, telomerase is composed of two subunits: The catalytic subunit named human telomerase reverse transcriptase (hTERT) and the telomerase RNA component (TERC) for the de novo synthesis of telomeric DNA sequences. The TERT gene, located on the chromosomal area 5p15.33, is the primary regulator of TA via its core promoter region and numerous binding sites which all together serve as transcription regulators. In fact, the main regulatory checkpoint of TA is at its transcription [44]. However, following the genes’ pathway upstream, it can be seen that TERT expression is regulated by a number of transcription factors, including c-Myc, Mad1, estrogen receptor, progesterone receptor, AP-1, NF-kB, Rb/E2F factors, CEBP-alpha, and CEBP-beta [83,84] with the Wnt/beta-catenin pathway and the KLF4 being promising candidates as well [85,86]. Of note, it has been shown that TA decreases at the late stages of in utero life, while during ex utero life, it is almost diminished, namely in adult somatic cells [84]. However, an increasing body of evidence supports the notion that most types of cancer cells, among which are skin cancer cells, exhibit an increased TA, mainly due to TERT promoter mutations [87]. Surprisingly, it has also been described that mutations of the TERT gene are of paramount importance for cancer cells derived from tissues with low rates of cellular regeneration [88]. Studying the various TA profiles in skin cancer, Parris I reported that patients with skin cancer exhibited a higher TA than the healthy controls, regardless of the type of cancer. Moreover, a difference in TA was witnessed between the various subtypes of NMSC. In detail, TA was increased in the majority of BD, AK and BCC cases, whereas only in a small number of SCC patients (25%, 3/12). Another interesting finding was the gradual increase in TA in pre-cancerous lesions (42% of AK and BD cases, 11/26) to confirmed cancers (77% of the BCC patients, 10/13) [89]. On the contrary, Boldrini et al. examined a small series of SCCs and BCCs found that SCCs exhibited a higher TA than BCCs, while a close association between hTERT expression and TA was also found. That is of utmost importance, given the relative simplicity of RT-PCR in contrast to TRAP-ELISA, which is the test mostly used for the determination of TA [90]. In a series of 66 patients with NMSC (32 with BCC and 34 with SCC), Griewank et al. found that approximately 50% of both groups had TERT promoter mutations accompanied by significant UV damage in their DNA, with no statistically significant association found with clinicopathologic parameters [91]. In accordance with these findings, Scott et al. reported that TERT promoter mutations were present in 18/23 sporadic BCCs (78%), 13/19 BCCs with nevoid basal cell carcinoma syndrome (68%), 13/26 SCCs (50%), and 1/11 BDs (9%) from a total of 18, 4, 19, and 11 patients, respectively, while being absent in their control group [92]. A finding that has to be noted is that each lesion bears its own genetic fingerprint. That is of utmost importance in cases with multiple lesions where an error in a sampling test should be avoided.

### 3.3. Epigenetic Modifications

Eukaryotic cells may be subsequent to heritable and non-heritable genomic alterations. Heritable genomic alterations that are not produced by changes in the genomic DNA sequence are summarized as epigenetics [93]. Epigenetic modifications include DNA methylation of the C-5 position of the cytosine ring within the promoter’s CpG island, histone methylation and acetylation, and miRNA-mediated gene regulations. Separately and combined, these alterations regulate the chromatin formation and packaging and thus regulate gene transcription by modifying their accessibility [94]. It is accepted that epigenetic modifications reflect the environmental burden of an organism through its exposure to various toxicants and carcinogens [95].

#### 3.3.1. CpG Island Methylation (CIM)

DNA methylation is one the most important regulatory mechanisms for gene expression. In normal cells, it assures the proper regulation of gene expression and stable gene silencing. This is achieved through the recruitment of DNA methyltransferases (DNMTs) in order to introduce methyl groups in cytosine within CpG dinucleotides by creating covalent bonds between them. In fact, CpG dinucleotides may appear in large clusters known as CpG islands (Figure 1). Intense research in cancer biology has identified global genomic hypomethylation as one of the leading factors for genomic instability and oncogene activation, whereas a number of tumor suppressor genes are silenced due to hypermethylated CpG islands [96], while global hypomethylation of lamina-associated domains (LAD) may be another aspect of the deregulated methylome [97]. In cutaneous melanoma, it was demonstrated that promoter hypomethylation and intragenic hypermethylation of specific genes are associated with tumor aggressiveness due to the alteration of extracellular matrix components and the upregulation of matrix metalloproteinases [98,99,100]. This highlights the clinical potential of deregulated methylation status as a hallmark for carcinogenesis, allowing the recognition of various methylation patterns as biomarkers for diagnosis and prognosis [101] (Table 1). Methylation studies focusing on cSCC, have demonstrated various patterns. For instance, numerous promoters have been found to be hypermethylated, among which are the cell cycle regulator CDKN2A [102], cadherin CDH1 [103,104] and CDH13 [105], transcription factor FOXE1 [106], modulators of Wnt signaling SFRPs [107] and FRZB [108], positive regulators of apoptosis ASC [109], G0S2 [110], DAPK1 [111], and miRNA-204 [112], as well as the hypomethylation of the DSS1 gene [113]. Hervás-Marín et al. compared low-risk and high-risk SCC and succeeded in identifying specific modifications of the methylation status using genome-wide DNA methylation profiling. In detail, they demonstrated a differential methylation status between the two pathological stages, with low-risk SCCs being hypomethylated and high-risk SCCs hypermethylated. According to the authors, this finding may suggest a sequential approach of SCC formation, where UV-exposure leads to hypomethylation and thus foretells the premalignant and low-risk stages of cSCC, while advanced stages of SCC present a hypermethylated status [101]. As regards the evaluation of the methylation status of BCC, Goldberg et al. presented the FHIT promoter to be hypomethylated [114], while Heitzer et al. found the hypermethylated PTCH promoter only in a small number of cases [115]. Darr et al. examined metastatic BCCs and SCCs compared to their non-metastatic counterparts. They found that both metastatic entities exhibited a differential methylation status from the non-metastatic ones with pronounced global hypomethylation, as well as at tumor suppressor genes and PRC2 target genes. Moreover, MYCL2 was specifically found to be demethylated in metastatic cases. Of note, the authors highlighted the resemblance between the methylation pattern of metastatic BCC and cSCC regardless of the metastatic capacity [108]. Greenberg et al., studying a series of MCCs, demonstrated that the tumor suppressor p14-ARK was hypermethylated [116]. Moreover, hypermethylated promoters have also been found in DUSP2, CDKN2A, and members of the RASSF family [117]. The concomitant analysis of overexpressed proteins derived from methylated genes and hallmark mutations of skin cancers through high-sensitive molecular techniques is representing a promising strategy for the early diagnosis of tumors and to define the prognosis of patients [118].

#### 3.3.2. Histone Methylation and Acetylation

Histones are a family of five basic proteins (H1/H5, H2A, H2B, H3, and H4) whose role is to react with DNA strands in the nucleus assisting its dense packaging into chromatin and chromosomes. Histones H2A, H2B, H3, and H4 form a reel of dimers (the octameric nucleosome core) around which DNA is wrapped, while histones H1/H5 link nucleosomes together, allowing for an even higher degree of density (Figure 1). A key feature of histones is the presence of the N-terminal tail regions, which are rich in lysine residues. The histone tails can undergo extensive modifications, including methylation, acetylation, phosphorylation, sumoylation, and uquitinylation [119,120]. However, acetylation and methylation are the most well-studied aspects of histone modification, particularly in the setting of cancer. The acetylation and deacetylation of lysine residues modifies the net positive charge (decreasing or increasing it accordingly). Furthermore, the introduction of acetyl-groups induces a decreased affinity between histones and DNA, allowing for various transcription factors to reach regulatory areas such as gene promoters, while deacetylation has the opposite effect on gene expression by increasing the affinity between DNA and the histone complex [116]. Histone acetylation and deacetylation are catalyzed by the specific enzymes, histone acetyltransferases (HATs) and histone deacetyltransferases (HDACs), respectively. Histones are mainly methylated on the lysine and arginine residues of H3 and H4 tails [93]. The introduction of methyl-groups increases the hydrophobicity of histone proteins, inducing their tighter packing and thus inhibiting DNA transcription. Notably, it has been described that the restoration of normal histone density (reduction of DNA methylation and increase of histone acetylation) allows for the reactivation of the silenced tumor suppressor genes Cip1/p21 and p16 [121]. Rao et al. investigated the activation status of EZH2 (a histone methyltransferase of the polycomb repressive complex 2) and its related proteins in the context of aggressive BCCs. EZH2 is closely associated with the Sonic Hedgehog pathway [122]. According to their findings, EZH2 was upregulated (as in other studies [123]), allowing for a stratification between pathological stages. On the contrary, upregulated H3K27me3 and 5hmC were positively associated with a more benign phenotype. Finally, the authors were able to discriminate BCCs from non-malignant epidermal cells through the upregulated levels NSD2, MOF, H3K27me3, and 5hmC [124]. Harms et al. investigated a series of MCCs and found that EZH2 was deregulated, inducing gene silencing via histone H3 lysine 27 trimethylation and was thus associated with unfavorable characteristics, such as disease progression and a poorer prognosis [117,125]. However, even though histone methylation/acetylation has been extensively investigated in melanoma [116,126], research on NMSCs is limited. Indeed, it was recently demonstrated that the methylation of H3K4 is associated with the neoplastic transformation of melanocytes that evolve into cutaneous melanoma [127]. These results suggest that the epigenetic modification of histones’ methylation status could represent a promising epigenetic therapy for melanoma and other tumors [126].

#### 3.3.3. MicroRNAs (miRNAs or miRs)

miRNAs are small single-stranded non-coding RNAs of 18–25 nucleotides length. Their discovery in 1993 from two research groups working on *Caenorhabditis elegans* proved to be a milestone of what is now considered a true breakthrough in molecular biology [128,129]. However, for a number of years, the properties miRNAs remained poorly understood. Surprisingly, miRNA production is a refined, multi-step process, where specific DNA transcripts produce primary miRNAs (pri-miRNAs), which are processed into precursor miRNAs (pre-miRNAs) and then into mature miRNAs. Mature miRNAs have the potential to target specific mRNAs, leading to their degradation or inhibiting their translation into proteins. This is possible either through an interaction with the 3′-untranslated region (3′ UTR) of the target mRNA (in which case its expression is inhibited) [130] or through binding with other regions, such as the 5′-untranslated region (5′ UTR), coding sequence and gene promoters [131]. Of note however, miRNAs are able to regulate not only protein translation, but also gene expression. In detail, miRNAs have been found to be able to positively regulate gene expression under certain conditions [132]. This is possible as miRNAs are able to move through different cellular compartments [133] (Figure 1). However, miRNAs are not restricted to the cytosol. A number of studies have demonstrated the presence of miRNAs in the extracellular compartment, both in a free state and packed in various carriers, such as high density lipoprotein particles, apoptotic bodies, and others [134]. Indeed, in addition to their small size and hairpin-loop structure, they are unreachable to the various free RNases, allowing them to maintain their structural integrity [135]. Thus, isolating them from a variety of clinical specimens is possible. Lastly, it has been well established that miRNAs are actively secreted by a variety of cancer cells into the circulation [73]. However, each type of cancer expresses different miRNAs; thus, in this manner, each type of cancer creates its own molecular profile. This is of utmost importance when considering miRNAs as biomarkers for monitoring cellular activity and the genomic/proteomic status. Even though miRNAs can be isolated both from tissue samples and from biological fluids (serum, plasma, and urine), circulating miRNAs are the first choice in the clinical setting. This is due to the fact that tissue miRNA sampling is an invasive technic lacking the ability to provide reproducible results regardless of the operator and area of sampling [136]. At present, several studies have identified sets of miRNAs specific for different tumors, including lung cancer, mesothelioma, bladder cancer, colorectal cancer, glioblastoma multiforme, oral cancer, uveal melanoma, hematological malignancies, etc. [137,138,139,140,141,142,143,144].

Regarding NMSCs, owing to the dominance of BCCs among all other tumor types, numerous studies have focused on the identification of potential miRNA markers. Sand et al. used next-generation sequencing of the basal cell carcinoma miRNome and succeeded in identifying a number of upregulated miRNAs, of which the 10 most increased were hsa-miR-223-3p, hsa-miR-197-3p, hsa-miR-342-3p, hsa-miR-505-3p, hsa-miR-204-5p, hsa-miR-941, hsa-miR-145-5p, hsa-miR-301b-3p, hsa-miR-452-5p, and hsa-miR-191-5p [145]. Yi et al. found that miR-203, a specifically expressed miRNA in the epidermis [146], is consistently downregulated in cases of BCC. Moreover, they proved that c-JUN suppressed miR-203, while miR-203 also targeted c-JUN, creating an inhibitory loop. In addition, miR-203 was further suppressed by the synergistic oncogenic activity of the Sonic Hedgehog and EGFR pathways. It is rather interesting that various studies have identified c-JUN as a potent oncogene, mediating its action downstream of the Sonic Hedgehog pathway [147]. Thus, a simultaneous activation of the Sonic Hedgehog and EGFR pathways, in addition to a potential crosstalk between them may result in BCC formation. Given the inhibitory effect of miR-203 c-JUN, researchers have investigated the therapeutic potential of miR-203 administration. Indeed, high levels of miR-203 have been shown to result in a decreased c-JUN and p63 expression, indicating the effective suppression of target genes [148]. Hu et al. examined 86 patients with BCC in order to explore the association between the expression level of miR-34a in serum and the clinical prognosis. According to their findings, patients with BCC exhibited lower miR-34a levels compared to healthy controls. Data analysis further revealed that miR-34a was upregulated in cases with a larger tumor diameter, the absence of lymph node infiltration and non-invasive disease. Moreover, miR-34a was positively associated with various survival parameters, such as median progression-free survival, median overall survival, and the overall survival rate. However, no association was found with pathological staging or the primary site. On the contrary, cases with a profound downregulation of miR-34a presented a poor prognosis [149].

In SCC, numerous miRNAs have been found to be dysregulated. Some of these (namely miR-21, miR-205, miR-365, miR-31, miR-135b, miR-424, miR-320, miR-222, miR-15a, miR-142, and miR-186) have been shown to possess carcinogenic properties by targeting key genetic modulators, such as the PTEN, PDCD4, GRHL3, HOXA9, and RhoBTB genes or the AKT/mTOR pathway [150,151]. There is sufficient evidence to indicate that these genes are involved in crucial carcinogenic steps, such as tumor growth, invasion, migration, the maintenance of stem cell properties and the evasion of apoptosis [151]. On the contrary, there is a wide panel of carcinoprotective miRNAs (miR-20a, miR-203, miR-181a, miR-125b, miR-34a, miR-148a, miR-214, miR-124, miR-204, and miR-199a), which have been found to regulate genes, such as HMGB1, SIRT6, MMPs, MAP kinases, KRAS, LIMK1, c-MYC, SHP2, CD44, BCAM, FZD6, DDR1, and ERKs. The potential action is described to be via the regulation of the cell cycle, epithelial–mesenchymal transition, and stemness, while they have also been found to promote cellular apoptosis and senescence [152]. A number of studies have evaluated the association of various miRNAs with clinocopathological features. miR-205 has exhibited an association with various pathological features of a poor prognosis, such as desmoplasia, perineural invasion and infiltrative patterns, while clinically it has been linked to local recurrence [153,154]. Recently, Gong et al. described that miR-221 also has carcinogenic properties. This is achieved as miR-221 has been found to interact with PTEN, which is a key oncogene. Notably, the authors pinpointed the potential development of anti-miR-221 antibodies, assisting both diagnosis and treatment [155]. On the contrary, miR-203 expression was shown to be associated with a favorable prognosis, as it was primarily found in well-differentiated zones only and rarely in the invasion front [153]. Zhang et al. found that SCC patients with low miR-20a levels exhibited a significantly poorer overall survival than those with a high miR-20a expression. Moreover, miR-20a expression was closely associated with the TNM stage, as it was proven that a low level of miR-20a expression was more frequently exhibited in tumors with an advanced TNM stage [156]. Several studies have also examined the expression profiles of various miRNAs in MCC. Ning et al. used next-generation sequencing of small RNA libraries on tissue samples and identified the MCC miRNome. In total, eight miRNAs were overexpressed (miR-502-3p, miR-9, miR-7, miR-340, miR-182, miR-190b, miR-873, and miR-183) and three miRNAs were suppressed (miR-3170, miR-125b, and miR-374c) in contrast to other forms of NMSCs. In situ hybridization further proved that miR-182 was abundant within cancer cells. The concomitant evaluation of the expression profiles of four miRNAs (miR-182, miR-183, miR-190b, and miR-340) in the MCPyV-negative cell line, MCC13, proved that they were downregulated. Thus, they proposed the possible diagnostic use of this miRNA panel in cases of MCPyV-positive MCC [157]. Veija et al. compared the miRNAome between MCPyV-positive and MCPyV-negative MCCs. According to their findings, miR-30a, miR-34a, miR-142-3p, and miR-1539 were overexpressed (2.5 to 5 times) in MCPyV-positive MCCs, while miR-181d exhibited a 3.5-fold higher expression in MCPyV-negative MCCs [158]. Renwick et al. used miRNA FISH in formalin-fixed paraffin-embedded tissues and succeeded in correctly discerning BCC from MCC, based on the overexpression of miR-205 and miR-375, respectively [159]. An important finding also derived from the study by Moens et al. who evaluated the secretion of various miRNAs in exosomes using RT-PCR. They succeeded in identifying the presence of miR-30a, miR-125b, miR-183, miR-190b, and miR-375 in exosomes [160]. This finding highlights the clinical potential of circulating miRNAs as biomarkers for MCC. In this context, the analysis of circulating tumor DNA and circulating miRNAs has been translated into clinical practice to predict the clinical-pathological features of tumors thus ameliorating the diagnostic and therapeutic strategies available for cancer patients [161]. However, solid evidence for the clinical relevance of extracellular miRNAs is still lacking. Moreover, despite the fact that numerous studies emerge daily, enriching the MCC-related miRNA panel, only a few of these have compared the expression profiles between malignant and non-malignant Merkel cells, and even fewer have tested the clinical or pathological relevance of these profiles [160,162]. miRNAs are recognized also as important biomarkers for the management of cutaneous melanoma. In this context, several studies have identified sets of miRNAs strictly associated to the development and progression of melanoma. In particular, Tao and colleagues (2019) have identified five miRNAs (miR-25, miR-204, miR-211, miR-510, and miR-513c) associated with survival of melanoma patients [163]. In the same manner, Hanniford et al. have identified a 4-miRNA signature (miR-150–5p, miR-15b-5p, miR-16–5p, and miR-374b-3p) predictive for the development of melanoma brain metastases [164]. Notably, some miRNAs, in particular the miR-510, are associated with both melanoma clinical features. A summary of the various miRNA expressions patterns and their clinical significance for NMSCs is presented in Table 2.

## 4. Biomarkers under Evaluation

### Micronuclei Frequency (MNf)

Micronuclei (MN), or Howell–Jolly bodies, are small cytoplasmic formations unsheathed in a nuclear envelope. In nature, they represent acentric chromatid/chromosome fragments (as a result of DNA damage) or whole chromatids/chromosomes (due to mitotic spindle failure, kinetochore damage, centromeric DNA hypomethylation and defects in the cell cycle control system) that are not included in the nucleus during telophase. Instead, they form small DNA-containing structures that are just a fraction of the size of the nucleus [165,166]. A large number of studies have indicated the promising potential of MN frequency (MNf) as a biomarker for diagnostic, prognostic and predictive use in various types of cancer, among which are those of the lung, bladder, and colorectal cancer [167,168]. However, both melanoma and NMSC have not been extensively studied with regards to their MNf status. Nonetheless, there is evidence that in premalignant cell lines (for example keratinocytes), MNf is higher than in normal skin lines [169], while chromosomal aberrations due to UVA and UVB skin exposure also result in an increased MNf [170,171]. Taking all these findings into consideration, it can be hypothesized that MNf as part of a wider panel of biomarkers, can be used not only for the diagnosis of NMSC, but also for a close and convenient monitoring for the early detection of tumor regression or progression.

## 5. Conclusions

NMSC is the most common type of cancer worldwide, representing an immense burden for both patients and healthcare systems. However, if diagnosed in an early stage, a great number of these cases will probably have a definitive care. Moreover, the vast majority of NMSC cases have well-studied causative factors, allowing for the establishment of screening protocols meant for high-risk groups. On the contrary, it is suggested that the macroscopic examination of the skin largely fails to assist secondary prevention improvement. Thus, the introduction of more sensitive and specific modes of diagnosis is required. The present review aimed to systematically suggest that molecular biomarkers are able to achieve this goal. In fact, molecular biomarkers seem to be promising candidates, not only for early detection, but also for the achievement of the corner stone of effective care which is personalized medicine. Despite the fact that NMSCs are distinct entities, they have been proven to share some common features to a certain extent. The hypermethylated E-cadherin (CDH1) promoter and the deregulated expression profile of miR-203 are some of the BCC/SCC shared biomarkers. However, as presented above, even if current literature suggests the possible clinical significance of various molecular targets (micronuclei frequency, extracellular miRNAs, histone methylation/acetylation) solid evidence on this topic is still missing. This highlights the need for further validation first through in vivo and then through large cohort studies where panels of sensitive and specific biomarkers will be evaluated both for their ability to detect and for their availability to foretell the prognosis. Unfortunately, a great disadvantage of NMSC biomarkers is the inability to specifically locate a lesion that has not made itself clinically/macroscopically evident yet. Thus, research for biomarkers has to create panels that will be not only disease-sensitive/specific but also site-sensitive/specific and therefore being able to discern between different body regions or between skin and mucous membrane cancers.

## Figures and Tables

**Table 1:** NMSC-related genomic loci, their methylation status, and their effect on cellular level.

Gene Target	Methylation Status	Type of NMSC	Cellular Effect	Reference
CDKN2A	Hypermethylated	SCC	Cell cycle deregulation	Brown et al. [102]
CDH1	Hypermethylated	SCC	Cellular environment deregulation	Chiles et al. [103] Murao et al. [104]
CDH13	Hypermethylated	SCC	Cellular environment deregulation	Takeuchi et al. [105]
FOXE1	Hypermethylated	SCC	Modulator of Wnt signaling	Venza et al. [106]
SFRPs	Hypermethylated	SCC	Modulator of Wnt signaling	Liang et al. [107]
FRZB	Hypermethylated	SCC	Modulator of Wnt signaling	Darr et al. [108]
ASC	Hypermethylated	SCC	Deregulation of apoptosis	Meier et al. [109]
G0S2	Hypermethylated	SCC	Deregulation of apoptosis	Nobeyama et al. [110]
DAPK1	Hypermethylated	SCC	Deregulation of apoptosis	Li et al. [111]
miRNA-204	Hypermethylated	SCC	Deregulation of apoptosis	Toll et al. [112]
DSS1	Hypomethylation	SCC	Deregulated post-translational protein modification	Venza et al. [113]
Global DNA	Hypomethylation	SCC (benign)	Restricted genomic silencing	Hervás-Marín et al. [101]
Global DNA	Hypermethylation	SCC (aggressive)	Extensive genomic silencing	
FHIT promoter	Hypomethylated	BCC	Replication stress and DNA damage	Goldberg et al. [114]
PTCH promoter	Hypermethylated	BCC (small number of cases)	Deactivation of tumor suppressor genes	Heitzer et al. [115]
MYCL2	Hypomethylated	BCC (metastatic)	Activation of proto-oncogene	Darr et al. [108]
p14-ARK	Hypermethylated	MCC	Deactivation of tumor suppressor genes	Greenberg et al. [116]
DUSP2, CDKN2A promoter	Hypermethylated	MCC	Deactivation of tumor suppressor genes	Harms et al. [117]

**Table 2:** Deregulated microRNA expression profiles and their clinical relevance for NMSC.

miRNA	Expression Status	Type of NMSC	Possible Significance	Reference
hsa-miR-223-3p,	Upregulated	BCC	Diagnosis	Sand et al. [145]
hsa-miR-197-3p,				
hsa-miR-342-3p,				
hsa-miR-505-3p,				
hsa-miR-204-5p,				
hsa-miR-941,				
hsa-miR-145-5p,				
hsa-miR-301b-3p,				
hsa-miR-452-5p,				
hsa-miR-191-5p,				
miR203	Downregulated	BCC	Diagnosis, Therapy	Yi et al. [146]
miR-34a	Downregulated	BCC	Prognosis	Hu et al. [149]
miR-21,	Upregulated	SCC	Diagnosis	Mizrahi et al. [150], Yu et al. [151]
miR-205,				
miR-365,				
miR-31,				
miR-135b,				
miR-424,				
miR-320,				
miR-222				
miR-15a,				
miR-142				
miR-186				
miR-20a,	Downregulated	SCC	Diagnosis	García-Sancha et al. [152]
miR-203,				
miR-181a, miR-125b, miR-34a,				
miR-148a, miR-214,				
miR-124,				
miR-204,				
miR-199a				
miR-205	Upregulated	SCC	Diagnosis, Prognosis	Cañueto et al. [153], Stojadinovic et al. [154]
miR-221	Upregulated	SCC	Diagnosis, therapy	Gong et al. [155]
miR-203	varied	SCC	Prognosis	Cañueto et al. [153]
miR-20a	Varied	SCC	Prognosis	Zhang et al. [156]
miR-502-3p,	Upregulated	MCC	Diagnosis	Ning et al. [157]
miR-9,				
miR-7,				
miR-340				
miR-182,				
miR-190b,				
miR-873,				
miR-183				
miR-3170,	Downregulated			
miR-125b,				
miR-374c				
miR-182,	Downregulated in MCPyV-negative cell line			
miR-183,				
miR-190b,				
miR-340				
miR-30a	Upregulated	MCPyV-positive MCCs	Diagnosis	Veija et al. [158]
miR-190b,				
miR-142-3p,				
miR-1539				
miR-181d		MCPyV-negative MCCs		
miR-375	Upregulated	MCC	Diagnosis	Renwick et al. [159]
miR-30a,	Upregulated	MCC	Diagnosis	Moens group [160]
miR-125b,				
miR-183,				
miR-190b				
miR-375				

## Abbreviations

NMSC	non-melanoma skin cancer
BCC	basal cell carcinoma
SCC	squamous cell carcinoma
MCC	Merkel cell carcinoma
BD	Bowen’s Disease
AK	Actinic Keratosis
UV	Ultraviolet
β-HPV	β-Human papilloma virus
UVB	ultraviolet B
UVA	Ultraviolet A
EGFR	epidermal growth factor receptor
FGFR	fibroblast growth factor receptors
EBV	Epstein–Barr virus
MCPyV	Merkel Cell Polyomavirus
LTAg	large T-antigen
STAg	small t-antigen
TL	Telomere length
hTERT	human telomerase reverse transcriptase
hTR	human telomere RNA
TERC	telomerase RNA component
TA	telomerase activity
PBL	peripheral blood lymphocytes
CIM	CpG island methylation
DNMTs	DNA methyltransferases
LAD	lamina-associated domains
HATs	acetyltransferases
HDACs	histone deacetyltransferases
pri-miRNAs	primary miRNAs
pre-miRNAs	precursor miRNAs
3′ UTR	3′-untranslated region
5′ UTR	5′-untranslated region
MN	Micronuclei
MNf	Micronuclei frequency
